# Scope and Limitations of a Novel Synthesis of 3-Arylazonicotinates 

**DOI:** 10.3390/molecules17055924

**Published:** 2012-05-18

**Authors:** Omniya Sayed Zaky, Moustafa Sherief Moustafa, Maghraby Ali Selim, Awatef Mohamed El-Maghraby, Mohamed Hilmy Elnagdi

**Affiliations:** 1 Chemistry Department, Faculty of Science at Qena, South Valley University, P.O. Box 83523, Qena, Egypt; 2 Department of Chemistry, Faculty of Science, University of Kuwait, P.O. Box 5969, Safat 13060, Kuwait

**Keywords:** 3-oxo-3-phenyl-2-phenylhydrazonal, arylazonicotinic acid, pyridine, electrocyclization, heteroaromatics

## Abstract

The reaction of 3-oxo-3-phenyl-2-phenylhydrazonal with functionally substituted and heteroaromatic substituted acetonitrile to yield arylazonicotinic acid derivatives and 5-arylsubstituted pyridines was established. In some cases the produced nicotinates could not be isolated as they underwent thermally induced 6π-electrocyclization yielding polynuclear pyridine derivatives.

## 1. Introduction

**Scheme 1:**
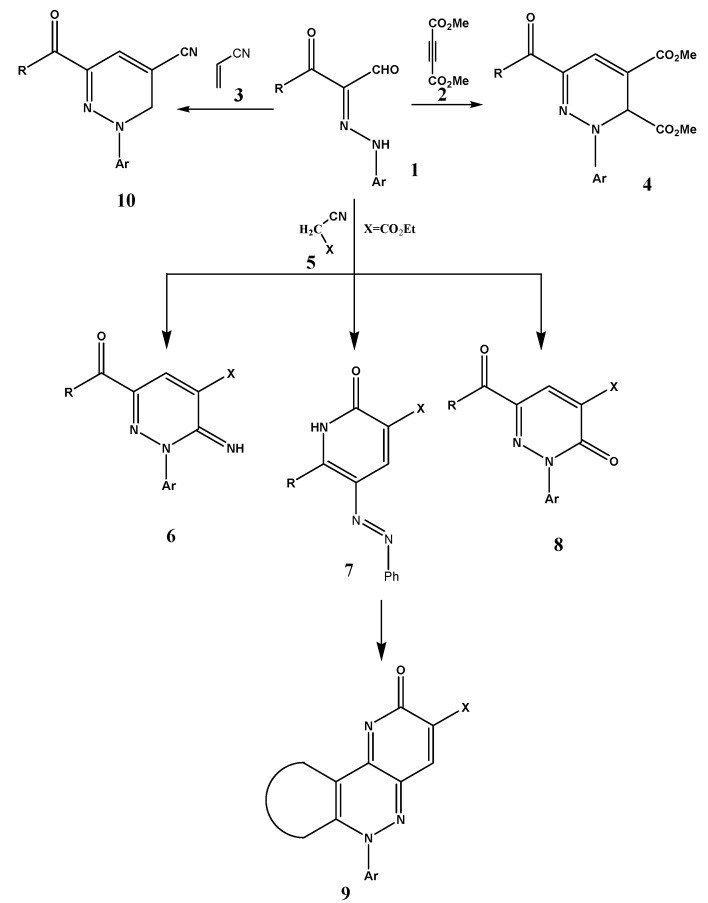
Chemical reactivies of 3-oxo-3-substituted-2-arylhydrazonals **1**.

**Figure 1:**
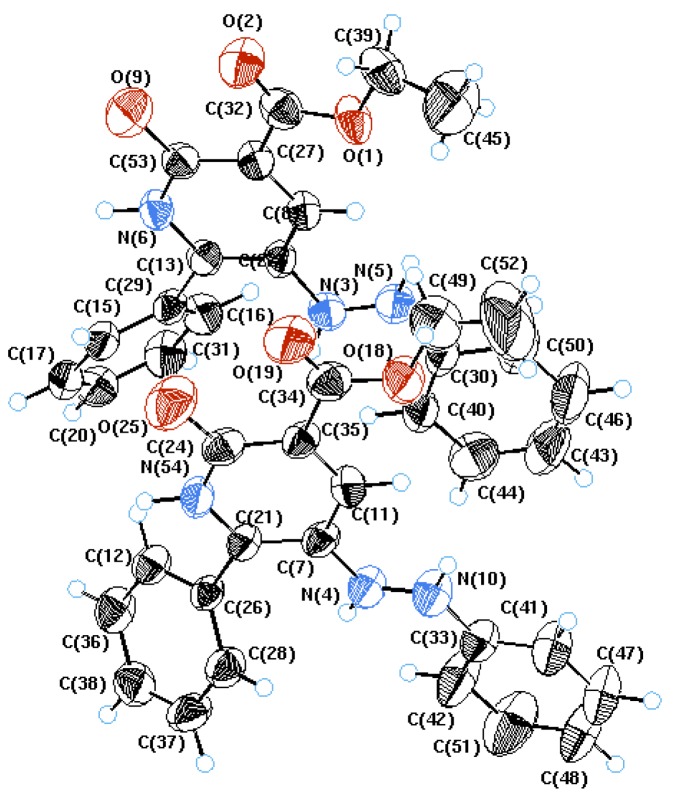
X-ray structure of compound **7**.

3-oxo-3-Substituted-2-arylhydrazonals **1** are versatile, readily obtainable reagents [1] that have been extensively utilized in the synthesis of polyfunctional substituted aromatics and heteroaromatics [2,3]. In the past we have reported novel synthesis of polyfunctional pyridazines **4** via heating **1** with dimethyl acetylenedicarboxylate (**2**) as well as acrylonitrile (**3**) in presence of triphenylphosphine or a tertiary amine base [4,5] (Scheme 1). We have also reported that condensing active methylene nitriles **5** with **1** affords products that were believed to be the pyridazine imines **6** [6]. Recently however Al-Mousawi *et al*. [7,8] realized that this structure cannot be correct as reported ^13^C-NMR data for the product lacked a carbonyl carbon at δ < 175 ppm. It was revealed that the products of condensing **1** with ethyl cyanoacetate are really the arylazonicotinates **7** (cf. Scheme 1), as clearly revealed by the X-ray crystal structure (Figure 1). 

However, in some case pyridazinones **8** were the reaction products rather than nicotinates. In light of the enormous potential of arylazonicotinates both as new pyridone dyes [9] and as biologically active compounds with anticonvulsant activity that act due to synaptic and non-synaptic mechanisms and some studies that have proved their antitumor and antimicrobial activities [10], we were interested in defininh further the behavior of **1** toward **5** to see if the reaction would afford **7** or **8**. In the present article we report on the reactivity of **1a–d** toward a variety of derivatives of **5** where we noted that this reaction may produce derivatives of **7** or **8** depending on the nature of **5**. Distinguishing between **7** and **8** could be easily accomplished based on ^13^C-NMR data as the absence of a carbonyl carbon signal would mean that the product is not an arylpyridazine derivative. Also with some derivatives of **7** electrocyclization and loss of hydrogen leading to novel 6-aryl-6H-pyrido[3,2-c]cinnolin-2-ones **9** seems probable.

## 2. Results and Discussion

**Scheme 2:**
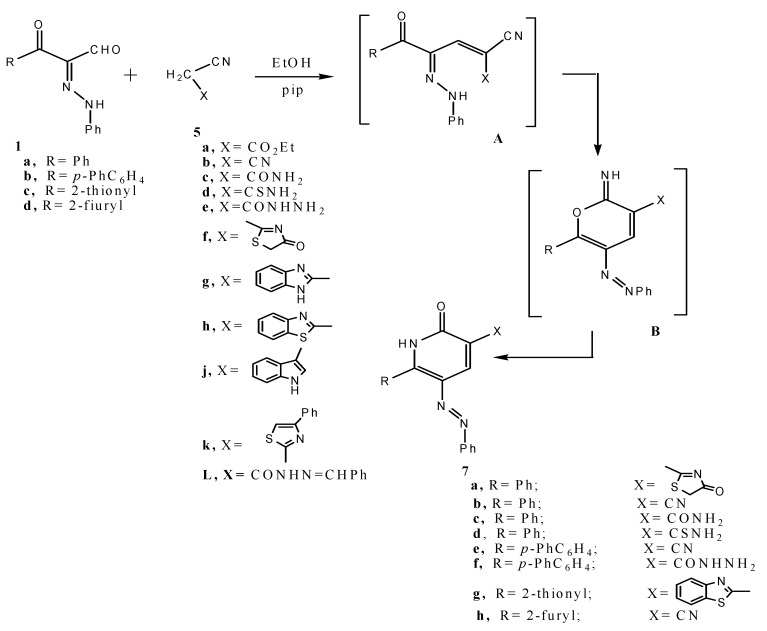
Syntheses of phenylazonicotinates **7a,c–g**.

Compounds **1a–d** reacted with malononitrile **5b** yielding the arylazonicotinonitriles **7b,h**, respectively as indicated by the absence of carbonyl carbon absorptions in the ^13^C-NMR of the products. Similar to their behavior toward malononitrile compounds, **1a–d** condensed with **5a,c–k** to yield phenylazonicotinates **7a,c–g** (Scheme 2).

**Scheme 3:**
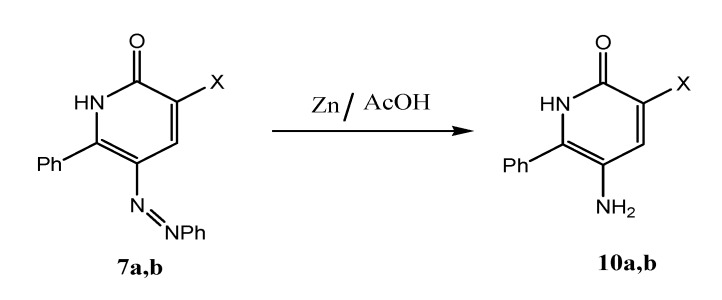
Syntheses of aminopyridinones **10a,b**.

Phenylazonicotinates **7a,b** were converted to aminopyridinones **10a,b** by reduction using Zn/AcOH (Scheme 3).

**Scheme 4:**
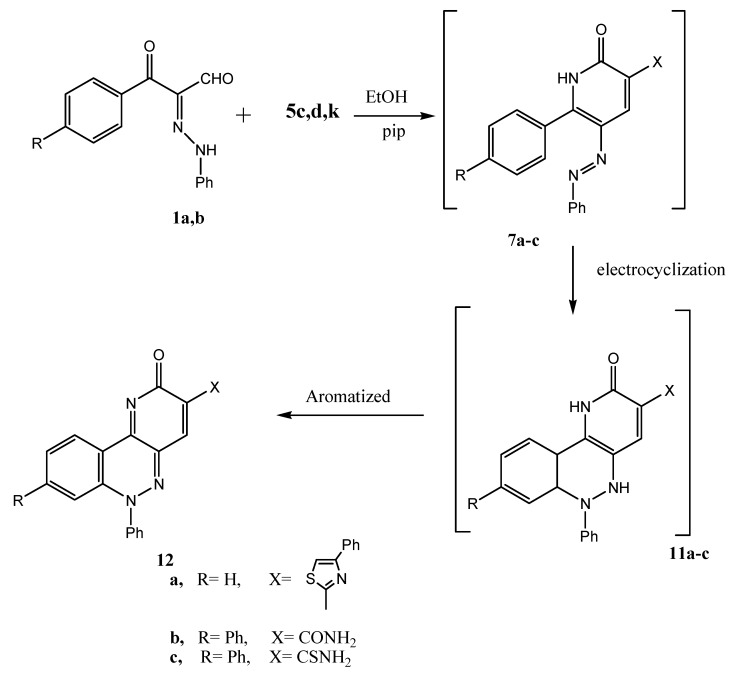
Syntheses of compounds **12a–c**.

The reaction of **1a,b** with **5c,d,k** in ethanolic piperidine solution resulted in the formation of **12a–c**. It is believed that the initially formed derivative of **7** readily underwent a 6π electrocyclization yielding **11a–c** that then aromatized to the final products **12a–c** (Scheme 4).

**Scheme 5:**
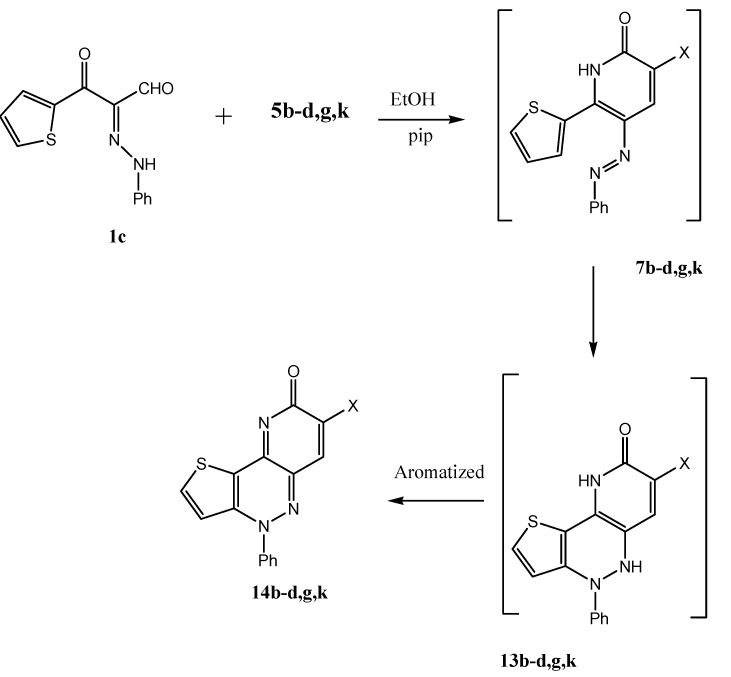
Syntheses of compounds **14b–d,g,k**.

We believe that the decreased aromaticity of the thiophene ring as compared to benzene is behind this ready electrocyclization, and in support of this conclusion we have found that **1c** also afforded **14b–d,g,k** upon reaction with **5b–d,g,k**; again the initially formed derivative of **7** underwent electro-cyclization to **13** and then aromatized to yield **14b–d,g,k** (Scheme 5).

**Scheme 6:**
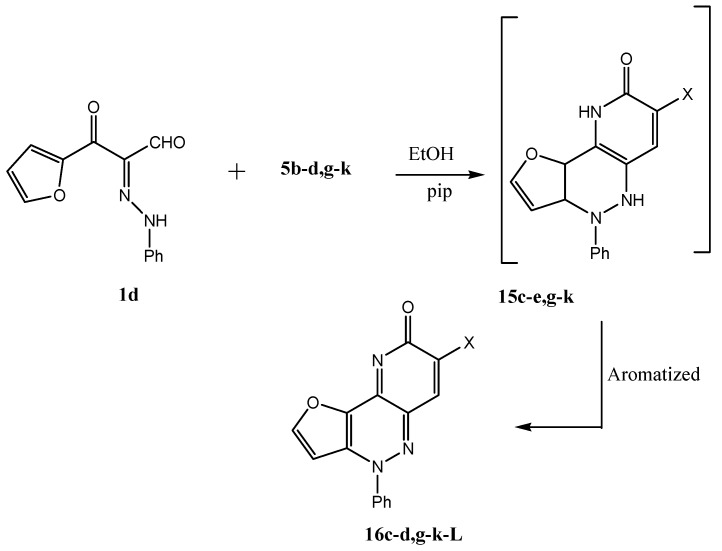
Syntheses of compounds **16c–e,g–k**.

Similar to this behavior compound **1d** reacted with **5c–e,g–k** to afford compounds **16c–e,g–k** under the same reaction conditions (Scheme 6).

In summary, we could clearly demonstrate that the structures of the products obtained by reacting **1a–d** with active methylenes can be readily concluded via inspection of position of the carbonyl carbon signals in the corresponding ^13^C-NMR spectra. When a arylhydrazone moiety in the intermediates cyclises via addition to a CN function subsequent hydrolysis of the formed imine usually occured leading to pyridazinones.

## 3. Experimental Section

### 3.1. General

Melting points are reported uncorrected and were determined with a Sanyo (Gallaenkamp) instrument. Infrared spectra were recorded using KBr pellets and a Perkin-Elmer 2000 FT-IR instrument. ^1^H- and ^13^C-NMR spectra were determined using a Bruker DPX instrument at 400 MHz for ^1^H-NMR and 100 MHz for ^13^C-NMR and DMSO*-d_6_* solutions with TMS as internal standard. Chemical shifts are reported in δ (ppm). Mass spectra were measured using a VG Autospec Q MS 30 and MS 9 (AEI) spectrometers, using the EI (70 EV) mode. Elemental analyses were carried out using a LEO CHNS-932 Elemental Analyzer

### 3.2. General Procedure for the Synthesis of Compounds ***7a,c–g***

A mixture of **1a–d** (0.01 mmol), and active methylenenitrile derivatives **5a–l** (0.01 mmol) in the presence of piperidine (5 drops) and ethanol (10 mL) as a solvent was refluxed for 1–2 h. The reaction mixture was evaporated. The solid product, so formed, was crystallized from a suitable solvent.

*3-(4-Oxo-4,5-dihydrothiazol-2-yl)-6-phenyl-5-phenylazo-1H-pyridin-2-one* (**7a**). Red crystals from ethanol, yield 95%; m.p. up 300 °С; Anal. Calcd. for C_20_H_14_N_4_O_2_S (374) calcd: C, 64.16; H, 3.77; N, 14.96. Found: C, 64.00; H, 3.54; N, 14.83; IR (KBr) υ_max_: 1,629 (CN), 1,670 (CO); ^1^H-NMR (DMSO-*d_6_*): δ = 1.3 (s, 2H, CH_2_); 7.0–8.1 (m, 10H, Ph-H); 10.0 (br, 1H, NH, D_2_O exchangeable); ^13^C-NMR (DMSO-*d_6_*): δ = 163.7, 162.9, 143.0, 137.0, 134.9, 129.0, 128.4, 127.7, 126.2, 124, 100.0, 39.0; MS: *m/z* (%) 373 (M^+^, 10), 299 (85), 224 (5), 140 (20).

*2-Oxo-6-phenyl-5-phenylazo-1,2-dihydropyridine-3-carbonitrile* (**7b**). Dark yellow crystals from ethanol, yield 95%; m.p. 153 °С; Anal. Calcd. for C_18_H_12_N_4_O (300): C, 71.99; H, 4.03; N, 18.66. Found: C, 71.80; H, 3.99; N, 18.53; IR (KBr): υ_max_: 3,264 (NH), 1,660 (CO); ^13^C-NMR (DMSO-*d_6_*): δ = 162.9, 156.9, 137, 134.9, 129.0, 128.4, 127.7, 126.2, 117.2, 106.7, 100.0; MS: *m/z* (%) 301 (M^+^, 50), 275 (20), 194 (15). 

*2-Oxo-6-phenyl-5-phenylazo-1,2-dihydropyridine-3-carboxylic acid amide* (**7d**). Orange crystals from ethanol, yield 98%, m.p. 190 °С; Anal. Calcd. for C_18_H_14_N_4_OS (334): C, 64.65; H, 4.22; N, 16.75. Found: C, 64.59; H, 4.21; N, 16.62; IR (KBr): υ_max_: 3,399–3,266 (NH_2_), 1,614(CN), 1,680 (CO); ^1^H-NMR (DMSO-*d_6_*): δ = 7.4–7.9 (m, 10H, Ph-H); 10.6 (br, 2H, NH_2_, D_2_O exchangeable); ^13^C-NMR (DMSO-*d_6_*): δ = 164.7, 135.7, 133.0, 130.3, 128.1, 126.4; MS: *m/z* (%) 334 (M^+^, 100), 105 (30), 77 (25).

*6-Biphenyl-4-yl-2-oxo-5-phenylazo-1,2-dihydropyridine-3-carbonitrile* (**7e**). Green crystals from AcOH, yield 95%; m.p. 145 °С; Anal. Calcd. for C_24_H_16_N_4_O (376.13): C, 76.58; H, 4.28; N, 14.88. Found: C, 76.57; H, 4.21; N, 14.62; IR (KBr) υ_max_: 3,343 (NH), 2,202 (CN), 1,655 (CO); ^13^C-NMR (DMSO-*d_6_*): δ = 162.0, 156.9, 137.0, 136.6, 135.8, 132.8, 129.0, 127.4, 126.7, 117.2, 106.7, 100.0; MS: *m/z* (%) 377 (M^+^, 90), 244 (20), 152 (50), 77 (30).

*6-Biphenyl-4-yl-2-oxo-5-phenylazo-1,2-dihydropyridine-3-carboxylic acid hydrazide* (**7f**). Buff crystals from ethanol, yield 95%; m.p. 237 °С; Anal. Calcd. For C_24_H_19_N_5_O_2_ (409): C, 70.40; H, 4.68; N, 17.10. found: C, 70.39; H, 4.61; N, 17.02; IR (KBr): υ_max_: 3,412–3,331 (NH_2_), 1,660 (CO); ^13^C-NMR (DMSO-*d_6_*): δ = 165.9, 162.9, 148.5, 137.0, 136.6, 135.8, 133.8, 131.3, 129.0, 127.4, 126.7, 100.0; MS: *m/z* (%) 409 (M^+^, 50) 324 (80), 181 (75), 77 (70).

*3-Benzothiazol-2-yl-5-phenylazo-6-thiophen-2-yl-1H-pyridin-2-one* (**7g**). Yellow crystals from ethanol/AcOH, yield 98%; m.p. 242 °С; Anal. Calcd. for C_22_H_14_N_4_OS_2_ (414): C, 63.75; H, 3.40; N, 13.52. Found: C, 63.71; H, 3.31; N, 13.42; IR (KBr): υ_max_: 3,264 (NH), 1,680 (CO); ^1^H-NMR (DMSO-*d_6_*): δ = 7.2–7.3 (t, 3H, thiol-H); 7.6–8.2 (m, 9H, Ph-H); 8.6 (br, 1H, NH, D_2_O exchangeable); 9.0 (s, 1H, nicotine-H);^13^C-NMR (DMSO-*d_6_*): δ = 162.9, 156.0, 153.0, 137.7, 136.6, 133.0, 130.0, 129.0, 127.8, 126.4, 125.0, 124.0, 123.0, 122.0, 106; MS: *m/z* (%) 413(M^+^, 100), 304 (25), 111 (40), 77 (10).

*6-Furan-2-yl-2-oxo-5-phenylazo-1,2-dihydropyridine-3-carbonitrile* (**7h**). Green crystals from ethanol, yield 95%; m.p. 214 °С; *Anal*. Calcd. for C_16_H_10_N_4_O_2_ (290): C, 66.20; H, 3.47; N, 19.30. Found: C, 66.19; H, 3.31; N, 19.30; IR(KBr): υ_max_: 3,322 (NH), 1,631 (CO); ^1^H-NMR (DMSO-*d_6_*): δ = 6.7–7.4 (m, 3H, furan-H); 7.6 (m, 2H, Ph-H); 8.1 (m, 2H, Ph-H); 8.1 (m, 1H, Ph-H); ^13^C-NMR (DMSO-*d_6_*): δ = 149.1, 148.7, 133.3, 130.4, 129.9, 126.1, 123.3, 112.8, 79.1; MS: *m/z* (%) 289 (M^+^, 100), 197 (5), 130 (5), 77 (50).

*Synthesis of 5-Amino-3-(4-oxo-thiazolidin-2-yl)-6-phenyl-1H-pyridin-2-one* (**10a**). A mixture of **7a** (3.6 g, 0.1 mol) and Zn powder (2 gm) in acetic acid (20 mL) was refluxed for 2 h. then filtered while hot. The reaction mixture was cooled to room temperature and then poured onto ice-water. The solid thus formed was collected by filtration and crystallized from AcOH to give black crystals, yield 70%; m.p. up 300 °С; Anal. Calcd. for C_14_H_13_N_3_O_2_S (287): C, 58.52; H, 4.56; N, 14.62. Found: C, 58.50; H, 4.54; N, 14.53; IR (KBr): υ_max_: 3,432, 3,213 (NH_2_), 1,658 (CO); ^13^C-NMR (DMSO-*d_6_*): δ = 164.0, 162.9, 134.9, 128.4, 127.7, 126.2, 121.0, 108.0, 51.4, 39.0; MS: *m/z* (%) 287 (M^+^, 50), 207 (10), 93 (65), 55 (40). 

*Synthesis of 5-Amino-2-oxo-6-phenyl-1,2-dihydropyridine-3-carbonitrile* (**10b**). A mixture of **7b** (0.1 mol) and and Zn powder (2 gm) in acetic acid (20 mL) was refluxed for 2 h. then filtered while hot. The reaction mixture was cooled to room temperature and then poured onto ice-water. The solid thus formed was collected by filtration and crystallized from AcOH to give pale brown crystals, yield 70%; m.p. 190 °С; Anal. Calcd. for C_12_H_9_N_3_O (211): C, 68.24; H, 4.29; N, 19.89. Found: C, 68.20; H, 4.19; N, 19.83; IR (KBr): υ_max_: 3,432, 3,312, (NH_2_), 1,640 (CO); ^13^C-NMR (DMSO-*d_6_*): δ = 162.0, 156.9, 134.9, 128.4, 127.7, 126.0, 121.1, 117.2, 108.0, 106.7; MS: *m/z* (%) 211 (M^+^, 10), 129 (25), 77 (80).

*6-Phenyl-3-(4-phenylthiazol-2-yl)-6H-pyrido[3,2-c]cinnolin-2-one* (**12a**). Deep red crystals from ethanol, Yield 98%; m.p. 150 °С; Anal*.* Calcd. for C_26_H_16_N_4_OS (432): C, 72.20; H, 3.73; N, 12.95. Found: C, 72.19; H, 3.61; N, 12.92; IR (KBr): υ_max_: 1,614 (CN), 1,680 (CO); ^1^H-NMR (DMSO-*d_6_*): δ = 7.2 (s, 1H, thiazole-H); 7.3–8.1 (m, 14H, Ph-H); 8.3 (s, 1H, nicotine-H); ^13^C-NMR (DMSO-*d_6_*): δ = 157.2, 154.9, 149.2, 141.1, 140.6, 136.1, 134.3, 133.3, 133.2, 131.1, 130.8, 130.7, 130.1, 129.3, 128.8, 128.5, 127.0, 126.6, 121.3, 120.9; MS: *m/z* (%) 433 (M^+^, 100), 329 (10), 105 (20), 77 (15).

*2-Oxo-6,8-diphenyl-2,6-dihydropyrido[3,2-c]cinnoline-3-carboxylic acid amide* (**12b**). Red crystals from ethanol, yield 90%; m.p. 230 °С; Anal*.* Calcd. for C_24_H_16_N_4_O_2_ (392): C, 73.46; H, 4.11; N, 14.28. Found: C, 73.35; H, 4.00; N, 14.11; IR (KBr): υ_max_: 3,267, 3,189 (NH_2_); ^13^C-NMR (DMSO-*d_6_*): δ = 165.0, 150.0, 148.9, 144.2, 139.8, 136.6, 130.0, 129.4, 127.4, 119.5, 118.0, 117.0; MS: *m/z* (%) 393 (M^+^, 100), 181 (75), 77 (50).

*2-Oxo-6,8-diphenyl-2,6-dihydropyrido[3,2-c]cinnoline-3-carbothioic acid amide* (**12c**). Orange crystals from AcOH, yield 95%; m.p. 170 °С; Anal. Calcd. for C_24_H_16_N_4_OS (408): C, 70.57; H, 3.95; N, 13.72. Found: C, 70.49; H, 3.71; N, 13.52; IR (KBr): υmax 3,399, 3,298 (NH_2_), 1670 (CO); ^13^C-NMR (DMSO-*d_6_*): δ = 164.0, 155.0, 144.0, 143.2, 141.0, 139.8, 136.6, 130.6, 129.0, 127.0, 119.5, 118.0, 116.9; MS: *m/z* (%) 407 (M^+^, 25), 391 (50), 151 (40), 51 (50).

*8-Oxo-4-phenyl-4,8-dihydro-1-thia-4,5,9-triazacyclopenta[a]naphthalene-7-carbonitrile* (**14b**). Yellow crystals from ethanol, yield 97%; m.p. 210 °С; Anal. Calcd. for C_16_H_8_N_4_OS (304): C, 63.15; H, 2.65; N, 18.14. Found: C, 63.05; H, 2.52; N, 18.11. IR (KBr): υ_max_: 1,640 (CO); ^1^H-NMR (DMSO-*d_6_*): δ = 7.2–7.5 (m, 2H, thiazole-H); 7.6–7.7 (m, 5H, Ph-H); ^13^C-NMR (DMSO-*d_6_*): δ = 141.4, 138.7, 138.4, 136.8, 136.9, 135.8, 134.9, 132.7, 131.7, 129.7, 129.0, 128.2, 127.8, 126.1, 117.2, 79.16; MS: *m/z* (%) 305 (M^+^, 80), 195 (5), 83 (15), 77 (25).

*8-Oxo-4-phenyl-4,8-dihydro-1-thia-4,5,9-triazacyclopenta[a]naphthalene-7-carboxylic acid amide* (**14c**). Orange crystals from ethanol, yield 98%; m.p. 270 °С; Anal. Calcd. for C_16_H_10_N_4_O_2_S (322): C, 59.62; H, 3.13; N, 17.83. Found: C, 59.59; H, 3.11; N, 17.80. IR (KBr): υ_max_: 3,400, 3,312 (NH_2_), 1,615 (CN), 1,680 (CO); ^1^H-NMR (DMSO-*d_6_*): δ = 7.2–7.2 (t, 2H, thiol-H); 7.6–8.0 (m, 5H, Ph-H); 8.1 (S, 1H, nicotine-H); ^13^C-NMR (DMSO-*d_6_*): δ = 147.5, 139.3, 139.0, 138.1, 137.2, 136.2, 133.3, 133.0, 130.3, 129.9, 128.8, 128.2, 126.1, 115.0, 114.2; MS: *m/z* (%) 323 (M^+^, 100), 306 (15), 111 (90), 77 (30).

*8-Oxo-4-phenyl-4,8-dihydro-1-thia-4,5,9-triazacyclopenta[a]naphthalene-7-carbothioic acid amide* (**14d**). Brown crystal from ethanol/AcOH, yield 90%; m.p. 195 °С; Anal. Calcd. for C_16_H_10_N_4_OS_2_ (338): C, 56.79; H, 2.98; N, 16.56. Found: C, 56.65; H, 2.82; N, 16.41; IR (KBr): υ_max_: 1,640 (CO), 1,620 (CN); ^13^C-NMR (DMSO-*d_6_*): δ = 164.15, 146.7, 144.0, 141.0, 129.3, 127.0, 126.0, 118.5, 115.1; MS: *m/z* (%) 339 (M^+^, 25), 111 (75), 77 (50).

*7-(1H-Benzoimidazol-2-yl)-4-phenyl-4H-1-thia-4,5,9-triazacyclopenta[a]naphthalen-8-one* (**14g**). Yellow crystals from ethanol, yield 95%; m.p. 230 °С; Anal. Calcd. for C_22_H_13_N_5_OS (395): C, 66.82; H, 3.31; N, 17.71. Found: C, 66.70; H, 3.21; N, 17.68; IR (KBr): υ_max_: 1,670 (CO), 1,620 (CN); ^1^H-NMR (DMSO-*d_6_*): δ = 7.2–7.2 (t, 1H, NH, D_2_O exchangeable); 7.2–7.3 (m, 2H, thiol-H); 7.5–8.1 (m, 9H, Ph-H); 8.4 (s, 1H, nicotine-H); ^13^C-NMR (DMSO-*d_6_*): δ = 151.9, 147.0, 138.5, 136.9, 136.0, 129.1, 128.9, 128.4, 128.1, 126.5, 123.3, 121.0; MS: *m/z* (%) 396 (M^+^, 100), 286 (25), 195 (15), 111 (90), 77 (30).

*4-Phenyl-7-(4-phenylthiazol-2-yl)-4H-1-thia-4,5,9-triazacyclopenta[a]naphthalen-8-one* (**14K**). Yellow crystals from ethanol, yield 95%; m.p. 200 °С; Anal*.* Calcd. for C_24_H_14_N_4_OS_2_ (438): C, 65.73; H, 3.22; N, 12.78. Found: C, 65.70; H, 3.11; N, 12.68; IR (KBr): υ_max_: 1,680 (CO), 1,620 (CN); ^1^H-NMR (DMSO-*d_6_*): δ = 7.2–7.4 (t, 2H, thiol-H); 7.4–8.1 (m, 10H, Ph-H); 8.41 (s, 1H, thiazole-H); 8.6 (s, 1H, nicotine-H); ^13^C-NMR (DMSO-*d_6_*): δ = 154.4, 140.2, 138.5, 137.0, 136.2, 133.8, 130.7, 130.3, 129.7, 128.9, 128.3, 128.1, 126.6, 126.1, 121.0, 119.52; MS: *m/z* (%) 439 (M^+^, 100), 368 (5), 236 (10), 111 (20).

*8-Oxo-4-phenyl-4,8-dihydro-1-oxa-4,5,9-triazacyclopenta[a]naphthalene-7-carboxylic acid amide* (**16c**). Yellow crystals from ethanol, yield 95%; m.p. 288 °С; Anal. Calcd. for C_16_H_10_N_4_O_3_ (306): C, 62.74; H, 3.29; N, 18.29.;Found: C, 56.40; H, 3.44; N, 16.32; IR (KBr): υ_max_: 1,685 (CO), 1,620 (CN); ^1^H-NMR (DMSO-*d_6_*): δ = 6.7–6.7 (m, 2H, furan-H); 7.0 (s, 1H, nicotine-H); 7.5–8.4 (m, 5H, Ph-H), 8.7 (br, 2H, NH_2_, D_2_O exchangeable); ^13^C-NMR (DMSO-*d_6_*): δ = 162.6, 149.0, 148.9, 139.9, 130.4, 129.8, 127.2, 126.5, 123.1, 112.7; MS: *m/z* (%) 307 (M^+^, 100), 290 (15), 95 (50), 77 (25).

*8-Oxo-4-phenyl-4,8-dihydro-1-oxa-4,5,9-triaza-cyclopenta[a]naphthalene-7-carbothioic acid amide* (**16d**). Deep brown crystal from ethanol, yield 95%; m.p. 220 °С; Anal. Calcd. for C_16_H_10_N_4_O_2_S (322): C, 59.62; H, 3.13; N, 17.38. Found: C, 59.40; H, 3.0; N, 17.3. IR (KBr): υ_max_: 1,638 (CO), 1,620 (CN); ^13^C-NMR (DMSO-*d_6_*): δ = 164.0, 155.0, 146.7, 143.0, 141.0, 129.3, 118.5, 115.1, 110.0; MS: *m/z* (%) 323 (M+, 25), 305 (75), 289 (60), 95 (80), 51 (40).

*7-(1H-Benzoimidazol-2-yl)-4-phenyl-4H-1-oxa-4,5,9-triaza-cyclopenta[a]n-aphthalen-8-one* (**16g**). Yellow crystals from ethanol, Yield 98%; m.p. 278 °С; Anal. Calcd. for C_22_H_13_N_5_O_2_ (379): C, 69.65; H, 3.45; N, 18.46. Found: C, 69.59; H, 3.41; N, 18.42; IR (KBr): υ_max_: 1,614 (CN), 1,670 (CO); ^13^C-NMR (DMSO-*d_6_*): δ = 151.9, 149.1, 148.7, 146.9, 138.8, 129.2, 128.4, 126.5, 123.0, 120.9, 112.7; MS: *m/z* (%) 380 (M^+^, 100), 286 (15), 195 (25), 95 (50), 77 (20).

*7-Benzothiazol-2-yl-4-phenyl-4H-1-oxa-4,5,9-triazacyclopenta[a]naphthalen-8-one* (**16h**). Orange crystals from ethanol, yield 98%; m.p. 258 °С; Anal. Calcd. for C_18_H_14_N_4_O_3_ (396): C, 66.6; H, 3.05; N, 14.13. Found: C, 66.59; H, 3.00; N, 14.12; IR (KBr): υ_max_: 1,614 (CN), 1,680 (CO); ^1^H-NMR (DMSO-*d_6_*): δ = 6.7 (s, 1H, furan-H); 7.3 (s, 1H, furan-H); 7.4–7.7 (m, 9H, Ph-H); 8.6 (s, 1H, nicotine-H); ^13^C-NMR (DMSO-*d_6_*): δ = 158.8, 151.4, 151.4, 149.0, 148.9, 148.8, 140.1, 140.0, 137.8, 131.0, 130.4, 129.8, 126.6, 126.6, 125.7, 123.3, 123.1, 122.1, 121.6, 112.8; MS: *m/z* (%) 397 (M^+^, 100), 303 (5), 212 (10), 95 (40), 77 (10).

*4-Phenyl-7-(4-phenylthiazol-2-yl)-4H-1-oxa-4,5,9-triaza-cyclopenta[a]naphthalen-8-one* (**16k**). Yellow crystals from ethanol, yield 98%; m.p. 230 °С; Anal. Calcd. for C_24_H_14_N_4_O_2_S(422): C, 68.23; H, 3.34; N, 13.26. Found: C, 68.19; H, 3.21; N, 13.12; IR (KBr): υ_max_: 1,614 (CN), 1,680 (CO); ^13^C-NMR (DMSO-*d_6_*): δ = 164.0, 155.0, 154.0, 146.7, 143.0, 139.0, 136.2, 129.3, 128.0, 127.0, 118.0, 115.1, 114.0, 110.0; MS: *m/z* (%) 423 (m^+^, 100), 329 (5), 238 (5), 95 (25), 77 (10).

*8-Oxo-4-phenyl-4,8-dihydro-1-oxa-4,5,9-triazacyclopenta[a]naphthalene-7-carboxylic acid benzylidenehydrazide* (**16l**). Orange crystals from ethanol, yield 98%; m.p. 266 °С; Anal. Calcd. for C_23_H_15_N_5_O_3_ (409): C, 67.48; H, 3.69; N, 17.11. Found: C, 67.45; H, 3.59; N, 17.00; IR (KBr): υ_max_: 1,559 1,614 (CN), 1,680 (CO); ^1^H-NMR (DMSO-*d_6_*): δ = 6.7 (s, 1H, CH); 7.2 (s,1H, nicotine-H); 7.4–7.5 (t, 2H, furan-H); 7.7–8.3 (m, 10H, Ph-H); ^13^C-NMR (DMSO-*d_6_*): δ = 130.5, 128.8, 127.4, 112.8, 106.4, 55.8, 18.9; MS: *m/z* (%) 410 (M^+^, 50), 291 (10), 105 (5), 77 (10).

## 4. Conclusions

In conclusion it has been found that **5** condenses with **1a** to yield pyridazinones **7** as indicted from the presence of a carbonyl carbon as δ = 165 ppm in the ^13^C-NMR. Initially formed imines **6** in this case are readily hydrolysed under the reaction conditions to yield the final products. In fact this finding supports our belief that heterocyclic imines like **6** are difficult to isolate as they readily afford the more stable aromatic derivative.

## Supplementary Materials

Supplementary materials can be accessed at: http://www.mdpi.com/1420-3049/17/5/5924/s1.
